# A Rare Case of a Giant Prostatic Urethral Stone and Its Management

**DOI:** 10.7759/cureus.81677

**Published:** 2025-04-03

**Authors:** Harsh Bhatia, Zeel Rakeshkumar Patel, Sofia Ali, Cosmas O Ihezie, Olasunkanmi A Kolawole, Mansi Singh

**Affiliations:** 1 Internal Medicine, Surat Municipal Institute of Medical Education and Research, Surat, IND; 2 Internal Medicine, Gujarat Medical Education & Research Society (GMERS) Medical College, Sola, IND; 3 Medicine, Peninsula Medical School, Plymouth, GBR; 4 Orthopedics, Federal Teaching Hospital, Owerri, Owerri, NGA; 5 Internal Medicine, University of Texas Health Houston School of Public Health, Dallas, USA; 6 Medicine, Bogomolets National Medical University, Kyiv, UKR

**Keywords:** benign prostatic hyperplasia (bph), clean intermittent self-catheterization (cisc), digital rectal examination, dysuria, holmium-yag laser lithotripsy, lower urinary tract symptoms (luts), prostatic calculi, prostatic stones

## Abstract

Prostate calculi, or prostatic stones, are typically small, greyish-brown, ultrasound-dense formations composed mainly of carbonated calcium phosphate apatite. We present a 65-year-old male with severe lower urinary tract symptoms (LUTS), including weak urinary stream, difficulty urinating, and acute retention, who was found to have a 4 cm prostate stone. Imaging confirmed obstruction of the prostatic urethra. Surgical intervention with transurethral resection of the prostate (TURP) and Holmium laser lithotripsy successfully removed the stone and relieved symptoms. This case emphasizes the need to consider prostate stones in severe LUTS when conventional treatments fail, with regular follow-up to monitor recurrence.

## Introduction

Lower urinary tract symptoms (LUTS) encompass a range of storage and voiding disturbances arising from dysfunction of the lower urinary system. LUTS are highly prevalent, affecting both men and women, with incidence increasing with age. Studies estimate that 50-70% of men over 50 experience LUTS, commonly due to benign prostatic hyperplasia (BPH), while over 40% of women report symptoms such as urgency, frequency, and incontinence, often linked to hormonal changes and pelvic floor dysfunction. The prevalence further rises with metabolic disorders, lifestyle factors, and socioeconomic disparities, making LUTS a significant public health concern.

LUTS can be categorized as storage-related (irritative) or voiding-related (obstructive) symptoms, reflecting the dynamic interplay between the bladder and urethral outflow system, particularly in men. Several factors have been implicated in the pathogenesis of LUTS, including age, hormonal changes, inflammation, lifestyle, metabolic diseases, congenital abnormalities, socioeconomic status, and nocturnal enuresis [1,2].

Age:Aging weakens bladder muscles, increases prostate size (in men), and reduces estrogen levels (in women), leading to incontinence.

Hormones: Estrogen deficiency weakens the pelvic floor, while testosterone influences prostate growth, contributing to LUTS.

Inflammation: Chronic inflammation (e.g., prostatitis, UTIs) sensitizes nerves and worsens symptoms.

Lifestyle: Obesity, smoking, and excessive caffeine/alcohol intake irritate the bladder and exacerbate LUTS.

Metabolic diseases: Diabetes and metabolic syndrome contribute to bladder dysfunction and oxidative stress.

Congenital factors: Conditions such as neurogenic bladder predispose individuals to lifelong LUTS.

Socioeconomic factors: Limited healthcare access, stress, and poor nutrition increase LUTS risk.

Nocturnal enuresis: It may indicate underlying bladder dysfunction or hormonal imbalance.

Understanding the multifactorial etiology of LUTS is essential for developing targeted interventions and improving patient outcomes. This study aims to explore the role of these risk factors in LUTS pathophysiology, providing insights into prevention and management strategies.

Prostatic stones are most often discovered incidentally during clinical and radiological examinations, such as when urological testing is conducted due to suspected abnormal prostate changes or through imaging techniques such as transrectal ultrasound or CT scan of the pelvis [3]. Despite advancements in diagnostic imaging and minimally invasive procedures, the rarity of giant prostate stones presents a distinct diagnostic and therapeutic challenge for urologists. We present the case of a 65-year-old male with severe LUTS, acute urinary retention, and an enlarged prostate with elevated PSA levels. Transrectal ultrasound and CT scan revealed a large prostatic calculus, an unusual finding.

## Case presentation

We present the case of a 65-year-old male with a history of severe LUTS, including difficulty urinating, a weak urinary stream, and frequent nocturia over the past six months. The patient came to the outpatient department (OPD) at Surat Municipal Institute of Medical Education and Research (SMIMER) hospital, Surat, India, for evaluation and management. He also reported intermittent lower abdominal pain and discomfort. The symptoms began gradually, with urinary hesitancy and a weak stream worsening over time. Frequent nocturia significantly disrupted his sleep, and he experienced occasional episodes of acute urinary retention, relieved by catheterization. Recently, he noted intermittent lower abdominal pain radiating to the perineum.

The patient's medical history included controlled hypertension and hyperlipidemia, with no history of urinary tract infections or kidney stones. His medication regimen included amlodipine and atorvastatin. Physical examination revealed an enlarged, firm prostate with irregular nodularity on digital rectal examination, but no tenderness.

**Figure 1:**
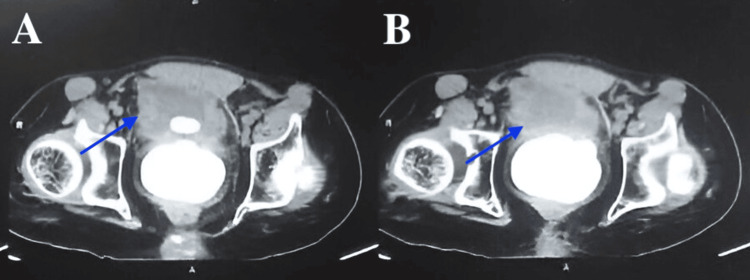
Non-contrast CT scan of the pelvis showing a massive prostate stone measuring approximately 4 cm within the prostatic urethra, causing significant obstruction (A, B). Blue arrows indicate the location of the stone within the urethra.

Laboratory investigations showed a normal complete blood count (CBC) and serum creatinine levels, but an elevated prostate-specific antigen (PSA) of 10 ng/mL. Imaging studies, including ultrasound of the kidneys and bladder, revealed mild bilateral hydronephrosis and significant post-void residual urine volume. A transrectal ultrasound (TRUS) identified a large, hyperechoic mass with posterior acoustic shadowing within the prostate, suggestive of a large prostate stone. A non-contrast CT scan of the pelvis confirmed the presence of a massive prostate stone measuring approximately 4 cm within the prostatic urethra, causing significant obstruction, as shown in Figure 1.

**Figure 2:**
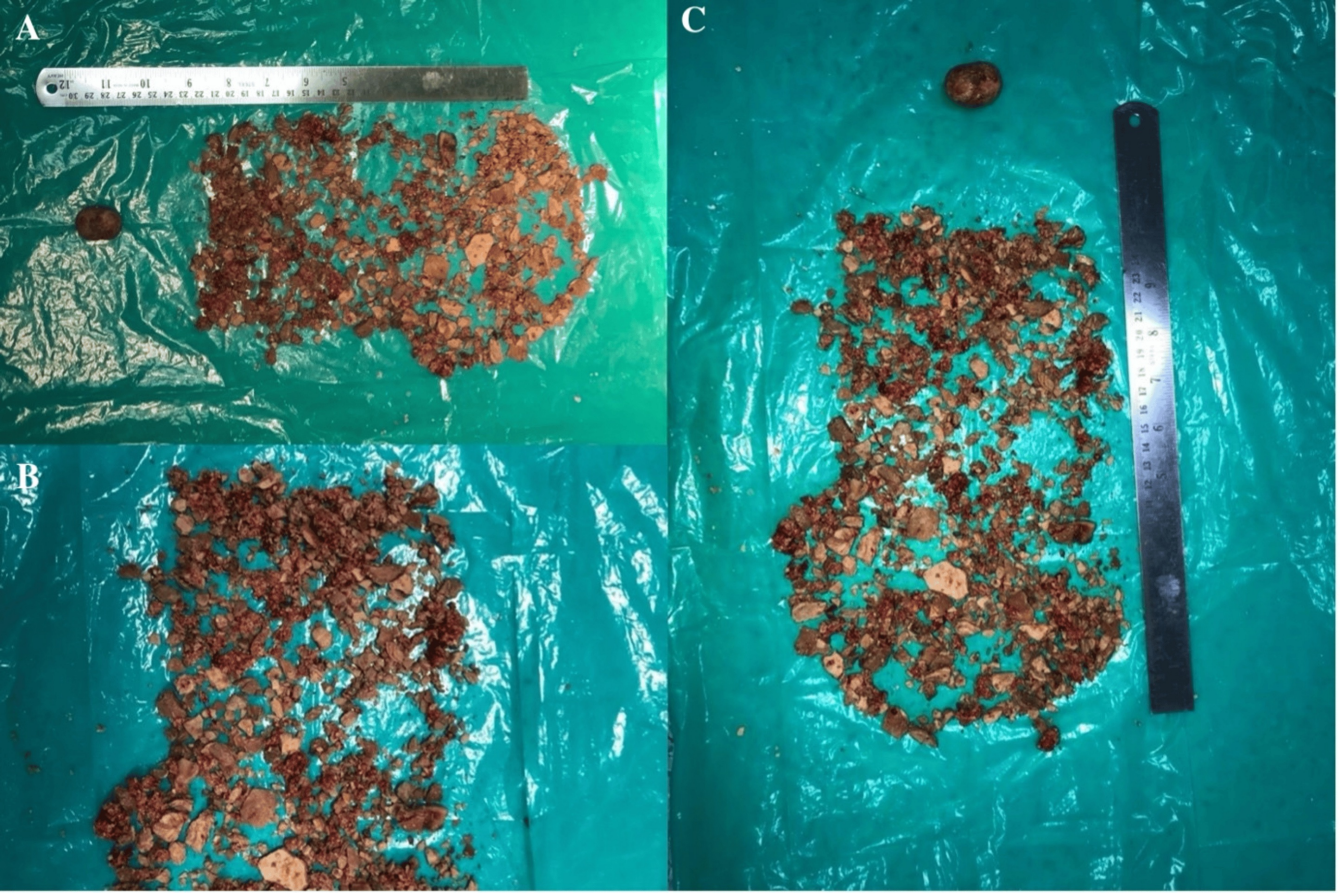
Specimen of the removed prostrate stone (A, B, and C).

The diagnosis was a massive prostate stone causing obstructive LUTS and intermittent acute urinary retention. Initial management involved catheterization to relieve acute urinary retention and pain management with analgesics. Given the stone's size and the severity of symptoms, surgical intervention was planned. A transurethral resection of the prostate (TURP) was performed to access and remove the prostate stone, which was fragmented using Holmium laser lithotripsy during the procedure. The removed stone was sent for analysis to determine its composition, as shown in Figure 2.

Postoperative care included monitoring for complications such as bleeding, infection, and urinary retention, administering prophylactic antibiotics to prevent infection, and continued catheterization for a few days to ensure proper drainage and allow healing. Follow-up involved monitoring PSA levels to check for any recurrence or underlying malignancy, a follow-up ultrasound to assess residual urine volume and kidney function, and regular visits to monitor symptoms and ensure no recurrence of obstruction. The patient underwent successful removal of the massive prostate stone via TURP and Holmium laser lithotripsy. Postoperative recovery was uneventful, with significant improvement in urinary symptoms. Regular follow-up is essential to monitor for recurrence and ensure optimal urological health.

## Discussion

LUTS affect up to 90% of men aged 45-80, with prevalence increasing with age across all racial and ethnic groups, peaking at 90% in men aged 81-90 [1,4]. It is classified into irritative (e.g., urinary frequency, urgency, nocturnal polyuria) and obstructive types (e.g., hesitancy, weak stream, incomplete emptying) [1]. However, other conditions, such as prostate stones, can also contribute to LUTS, with ongoing inconsistencies in research addressing these alternative causes. Prostate stones, often small and asymptomatic, can grow large and result in bothersome symptoms. They are common in patients with BPH, chronic pelvic pain syndrome, prostatitis, or following prostate radiotherapy. Prostatic stones have diverse chemical compositions, including carbonated calcium phosphate apatite, calcium oxalate, and magnesium [5-7].

Typically asymptomatic, prostate stones can present with urinary tract infections, prostatitis, dysuria, pain in the lower back, pelvis, scrotum, penis, or perineum, and urge incontinence, particularly at night. Prostate stones are most frequently observed in men from middle age onwards, with prevalence rates varying from 7% to 70% across different studies. In the healthy Han Chinese population, around 51.65% have prostate calcifications (PC), which increases to 76.61% in patients with BPH. The prevalence of PC also correlates with age, with rates of 9.0% in men aged 18-29 years, 32.3% in those aged 50-59, and 66.7% in the 70-79-year age group [8,9]. PC has been classified into types I and II (small lobular spheres and larger multifaceted surfaces), with Harada et al. further subdividing them into types A (discrete small reflections) and B (large multi-reflective masses) [10]. By crystallographic analysis, PC is categorized as endogenous, formed by prostatic secretions, or extrinsic, resulting from urinary reflux. Kodaka et al. divided prostatic calculi into four types based on formation mechanisms, with types I-III originating from the mineralization of amylaceous bodies and type IV from organic substances precipitating from prostatic secretions [6].

Prostate stones form through mechanisms such as urinary reflux, tissue degeneration, obstruction of secretory ducts, and inflammation, leading to calcification. Prostatic hypertrophy-induced chronic inflammation after the age of 50 contributes to stone formation, typically ranging from 0.5 to 5.0 mm in size. Despite inconsistencies in the formation of large prostatic stones, clinical complications are often underestimated by healthcare providers [2]. Tang et al. suggested that prostatic calcification may reflect a calcific environment in blood vessels [9].

This patient's clinical presentation, which included severe LUTS, intermittent acute urinary retention, mild bilateral hydronephrosis, and significant post-void residual urine volume, reflects mechanical obstruction caused by a large stone in the prostatic urethra. The absence of a urinary tract infection or nephrolithiasis makes this case unusual. Though the direct connection between kidney stones and prostate stones is limited, recurrent renal stones may increase the risk of stone formation elsewhere in the urinary tract. Nephrolithiasis, particularly in the presence of metabolic disorders such as hypercalciuria or hyperuricosuria, may predispose patients to stones in the prostate. However, BPH and chronic prostatitis are more commonly linked to prostate stones than nephrolithiasis. Urine collecting above an obstruction can harbor bacteria, increasing the risk of urinary tract infection [11,12].

Therapy, infection, trauma, inflammation, and BPH can elevate serum PSA levels. A PSA increase of 10 ng/mL raises concerns about possible underlying prostatic disease, including cancer. The prostate cancer risk is 25% for PSA levels between 4 and 10 ng/mL and around 50% when PSA levels exceed 10 ng/mL. PSA sensitivity varies from 9% to 33% based on age and cut-off values, suggesting that 25% of men managed by active surveillance may experience cancer progression requiring definitive treatment [13,14]. In this case, elevated PSA levels were attributed to the presence of the stone, with no underlying malignancy.

Diagnostic tools for prostatic calculi include gray-scale TRUS, which provides detailed images of the prostate and other pelvic organs and can detect prostatic calculi in both normal and abnormal prostates [10,15]. Serum bilirubin levels, influenced by vascular calcification risk factors such as smoking, triglycerides, and cystatin C, can predict prostatic calcification [9]. Digital rectal examination aids in diagnosing prostate stones, while TRUS offers a non-invasive, high-resolution, and immediate assessment of stone size and quantity [16]. X-ray imaging is less commonly used but can detect larger stones, though it is largely replaced by more advanced methods. CT provides precise cross-sectional views, helpful for determining stone location and size, particularly in complex cases [16]. MRI offers superior soft tissue contrast, which is useful for differentiating prostate stones from conditions such as tumors or abscesses [16]. In this case, a large, hyperechoic mass within the prostate with posterior acoustic shadowing was discovered by TRUS, suggestive of a massive prostate stone. A 4 cm stone obstructing the prostatic urethra was confirmed by non-contrast CT of the pelvis.

First-line treatment for relieving LUTS symptoms includes α-blockers, 5ARIs, analgesics, and PDE5 inhibitors, with ongoing research into the role of PDE5 inhibitors both independently and in conjunction with other therapies [4]. Clean intermittent self-catheterization (CISC) is the gold standard for managing urinary retention, defined as the repetitive temporary placement of a catheter to empty the bladder [17]. Surgical intervention is generally not needed for symptomatic prostatic calculi unless symptoms are severe. In such cases, total eradication is recommended. Prostatic calculi protruding into the urethra and causing retention can be removed through transurethral resection, which involves using a resectoscope loop to excise the stones and surrounding prostatic tissue. While transurethral resection offers relief, it may not always prevent the formation of new calculi [18].

Goyal et al. demonstrated that transurethral management using holmium-YAG laser lithotripsy is a safe and effective, minimally invasive technique for managing prostatic calculi of all sizes without morbidity [19]. A cystoscope or endoscope can also be used to remove stones via the urethra. If the prostate is enlarged during the procedure, prostate tissue may also be excised, a process called TURP [20]. In this case, analgesic pain management and catheterization were initially used to manage acute urinary retention. Surgery was planned due to the size of the stone and symptom severity. During TURP, the prostate stone was fragmented and removed using Holmium laser lithotripsy, with the stone sent for analysis to determine its composition.

This case highlights a rare differential diagnosis in obstructive uropathy, particularly when severe LUTS is accompanied by complexities that may require modifications to conventional treatments. This report discusses the patient’s clinical progression, diagnostic approach, and surgical success with TURP and Holmium laser lithotripsy and identifies gaps in current understanding of prostate calculi as a cause of severe LUTS, suggesting future directions for research on this condition.

## Conclusions

This case report highlights the significant impact of giant prostatic stones on LUTS. The 65-year-old patient experienced severe LUTS and acute urinary retention due to a 4 cm prostatic stone, which was effectively managed through transurethral resection and Holmium laser lithotripsy. This case demonstrates the importance of considering large prostatic stones in the differential diagnosis of LUTS, particularly when symptoms are severe or unresponsive to conventional treatments. The successful surgical intervention led to substantial symptom relief and underscores the need for careful evaluation and tailored management of such uncommon but challenging urological conditions.
